# Climate change impacts on health across the life course

**DOI:** 10.7189/jogh.14.03018

**Published:** 2024-05-24

**Authors:** Ruth A Etzel, Edda Weimann, Caroline Homer, Narendra Kumar Arora, Gloria Maimela, Elena Villalobos Prats, Anshu Banerjee

**Affiliations:** 1 George Washington University, Milken Institute School of Public Health, Washington, D. C., USA; 2 Technical University of Munich, Munich, Germany; 3 Burnet Institute, Melbourne, Australia; 4 INCLEN Trust International, New Delhi, India; 5 Wits Reproductive Health and HIV Institute, Johannesburg, South Africa; 6 World Health Organization, Department of Climate Change and Health, Geneva, Switzerland; 7 World Health Organization, Department of Maternal, Newborn, Child and Adolescent Health and Ageing, Geneva, Switzerland

## CLIMATE CHANGE IS AN IMPORTANT DETERMINANT OF HEALTH ACROSS THE LIFE COURSE

Climate change is transforming all aspects of life on earth, including human life. This issue of the journal includes three articles summarising the available reviews on the impact of climate change on maternal and newborn health, child and adolescent health, and older people, which together take a life course approach to optimal development and healthy ageing [1–3]. Reflecting evidence reviews, these articles explain that climate change has diverse and detrimental impacts on health and well-being across all life stages, from infancy to older age.

Climate change directly impacts health, such as the effects of extreme or sustained heat on susceptible sub-populations, including pregnant women, newborns, infants, children, vulnerable adults and older persons. It can also indirectly negatively affect health through multiple pathways by negatively affecting the underlying determinants of health. Examples include reduced crop outputs leading to food shortages, reduced nutritional intake, and decreased vitality (e.g. strength and metabolic balance). Climate change can increase the geographic range of disease vectors, such as mosquito species found at higher altitudes, leading to an increased incidence of malaria, West Nile fever, and Zika, among others, in new locations [4]. An increasing number of climate-induced disasters are experienced worldwide, including in small island states [5].

These and other direct and indirect effects are cumulative and occur across the life course. The effects of climate change can either be exacerbated by existing inequalities and the interaction with other crises or be mitigated through good governance, which draws on collective intelligence for the common good, evidence-based policies, the engagement of people, and the involvement of all sectors [6,7].

Good governance and health in all policies draw on the fundamental principle that all people have the right to health as part of human rights [8]. Moreover, all governments have committed themselves to achieving the Sustainable Development Goals [9], and most have signed the Paris Agreement [10]. These international policy instruments broadly underline that a) everyone’s basic needs, such as food, shelter and health services, must be met without discrimination, b) climate change and health are inextricably linked, and impacts are not confined to national borders, and c) importantly, policy choices can make a difference to anticipate, mitigate and adapt to climate change, if implemented at national, regional and global levels. Furthermore, evidence indicates that policies and actions will also need to address the social, economic, commercial and political determinants of climate change [11,12].

### Pathways describing climate change’s negative impact on the health of people at each life stage and across the life course

The articles in this special issue identify climate hazards and their differential impacts on health for three distinct life stages, discuss exposure pathways, and consider vulnerabilities at individual and population levels that put people at a higher risk. A framework for action must recognise the following key findings. A person’s context, genetic inheritance and social position will shape an individual’s vulnerability and resilience to climate change impacts. The accumulation of these risks identifies the most vulnerable life stages, including pregnant and postpartum women, children and adolescents, and older people, particularly those living in settings that do not provide protection from the vagaries of the changing climate.

Each life stage has common hazards such as extreme temperatures, wildfires, droughts, floods, storms and sea level rise; climate-sensitive infections; and poor air quality. Exposure pathways include food and water insecurity, displacement and migration, health and social systems impacts, and other critical services.

Measurable impacts are noted on all domains of health (e.g. physical, cognitive, psychological, sensory, vitality) across the life course. For example, psychological impacts include increased anxiety, depression, posttraumatic stress disorder and an increase in associated substance abuse disorders; air pollution can lead to reduced cognitive capacity, whether preventing optimal development for children or accelerating declines among older persons. Moreover, negative effects of climate change that occur during one stage of life impact subsequent life stages (**Box 1**).

Box 1 **Climate change impacts during pregnancy and infancy with lifelong consequences.**

Exposure during pregnancy to air pollution generated by burning fossil fuel, and extreme heat, is associated with hypertensive disorders, low birth weight, preterm birth, and affects foetal brain and lung development [13]. One meta-analysis concluded that the odds of preterm birth rose by 5% per 1°C increase in temperature and by 16% during heatwave vs non-heatwave days; therefore, there is greater risk with greater exposure and severity [14].

Even for full-term babies, climate change can have severe consequences on organs that are not fully developed at birth but grow rapidly in the first two years of life. Exposure to heat and air pollution can interfere with these developmental processes. For example, the brain develops faster in the early years than at any other time in life. Poor air quality and extreme heat can affect neurodevelopment with longer-term consequences for learning and productivity.

The lungs also grow rapidly in utero and continue developing after a child is born. During the first year of life, lung growth is exponentially fast, and exposure to air pollution adversely affects lung development [15]. Increases in respiratory infections and asthma linked to climate change, including fatalities, have been well documented [16]. Impaired development of organs and systems may have subtle or profound impacts on the child’s health and future life course, depending on the severity.

**Figure 1:**
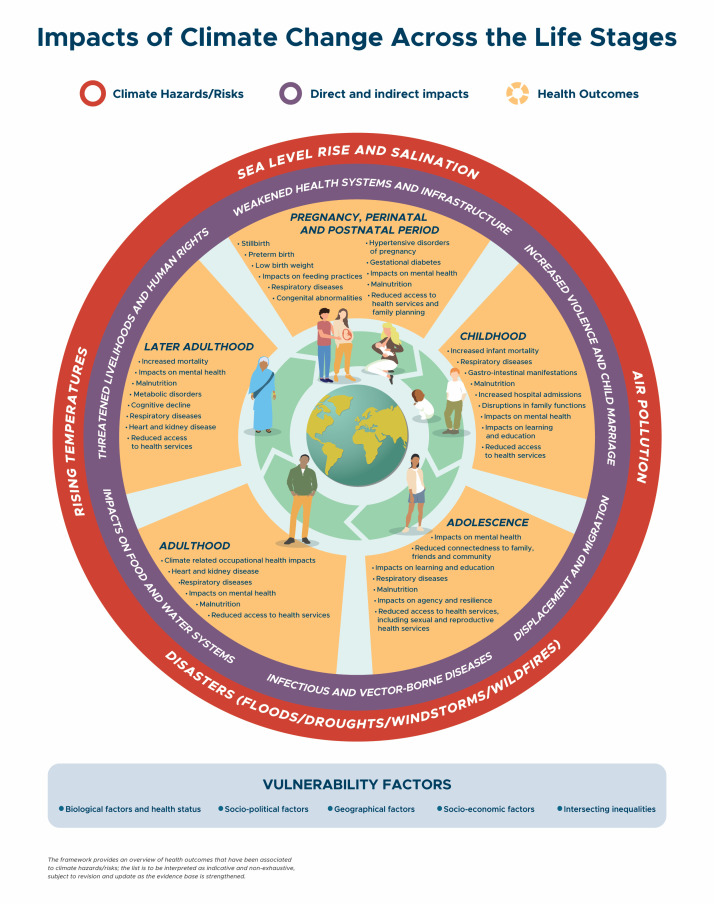
The impacts of climate change across the life stages. Vulnerability factors include: Biological factors and health status, sociopolitical factors, geographical factors, socio-economic factors, and intersecting inequalities.

Some hazards and exposure pathways are more marked or relevant for specific life stages due to critical or sensitive periods of each life stage (reflecting both biological and social aspects), combined with the specific geographic context, including the natural and built environment. For these reasons, studies are needed in diverse communities to assess actual impacts and exposure pathways (**Figure 1**).

Climate change also has significant effects on the mental health of women, children and adolescents, as it is associated with anxiety, depression, posttraumatic stress disorder and substance abuse disorders [17]. For example, maternal stress during natural disasters affects child development. Experiencing a natural disaster in early childhood has been linked to anxiety disorders in adulthood [18]. The climate crisis results in new disorders such as eco-anxiety and solastalgia. Older people also experience adverse brain effects from the changing climate; these include reduced cognitive function linked to air pollution, and increases in depression and posttraumatic stress disorder [19].

Further, 25 years ago, adverse childhood experiences were documented to have significant negative effects on adult physical and mental health, and a huge literature has accumulated in the past two decades confirming that they are linked to lifelong health effects [20,21]. Previously, few in the public health sphere considered exposures to climate change or contaminated natural environments as ‘adverse childhood experiences’. When the environment was mentioned, it referred to then-known adverse childhood experiences: exposures in the home to abuse and household dysfunction. The degradation of the natural environment (including climate change and toxic contamination) was largely invisible. Recently, however, the foundational importance of the natural environment has come into focus. The term ‘slow violence’ was coined by Rob Nixon to describe violence that ‘occurs gradually out of sight, with delayed effects, dispersed across time, typically not seen as violence’ [22]. Climate change is a superb example of slow violence, and the articles in this issue [1–3] suggest that experiencing the climate crisis constitutes an adverse effect, whether experienced in childhood or later in life.

### Urgent and comprehensive action is needed to address the root causes of health and other social inequities

Although there is increased awareness of climate change, action to safeguard the lives of those at most risk and vulnerable and improve the lives of all people is slow, if not imperceptible and fraught with injustices. On the one hand, the world is experiencing the boiling frog syndrome – if a frog is put suddenly into boiling water, it will leap out, but if the frog is put in tepid water that is then brought to a boil slowly, the frog will not perceive the danger and will be cooked to death. This is slow, yet violent.

On the other hand, limited options exist to combat this violence, particularly by those who are hardest hit, as evidenced by the situation of people in small island states and in other low-income countries who have contributed to less than 1% of the greenhouse gas emissions that caused the climate crisis [23]. Moreover, people living in poor conditions around the world have the least adaptive capacity to the impact of climate change on their health. For example, wealthy people usually have access to air conditioning and those living in poverty may not have a fan for cooling and often live in poorly designed houses that retain heat and expose them to air pollution or vector-borne disease. All people have the right to a healthy environment [24], and climate-resilient housing is a human right. There is an urgent need to build climate resilience, including enhancing preparedness for climate-related emergencies that can benefit all people, and to develop effective adaptation strategies for people at risk, with special attention to those living in poverty.

For climate action to be successful, meaningful engagement of people from across the social gradient and in all countries is imperative. Human ignorance about the importance of planetary health and the necessity for human activity to remain within ecological boundaries has caused this existential threat. According to the Intergovernmental Panel on Climate Change, because of the ongoing burning of fossil fuels, the world is not on track to keep global heating to under 1.5°C by 2030 [25,26].

The health inequities caused by climate change are shaped by upstream forces, including economics (greed), social policies (e.g. colourism, gender inequality) and political struggles. To address the sources of the climate crisis there is an immediate need for all countries to stop burning fossil fuels and adopt renewable sources of energy. Fiscal and economic policies, such as removing subsidies for fossil fuels, can incentivise a change in the behaviours of corporations and people. However, mitigation interventions, defined as actions and strategies aimed at reducing greenhouse emissions into the atmosphere, are insufficient and too slow. At the same time, the adverse impacts of burning fossil fuels must be addressed.

### Emerging strategies to anticipate, mitigate, and adapt, including improving equity

Three articles in this issue lead to a call for mitigation and adaptation interventions to protect health at each life stage, and specifically to incorporate each life stage and the unique risks and vulnerabilities of people into local and national climate change assessments, climate adaptation response plans and national policies [27–29].

From the health perspective, there are opportunities for health co-benefits from major mitigation strategies. There are many examples of positive strategies that benefit health and reduce greenhouse gas emissions. For instance, when we use active transport (walking or bicycling), burning calories instead of carbon, we do not burn fossil fuels and address obesity, especially among children [30,31]. When we have access to food that is fresh, local and lower on the food chain, (such as plant-based foods) we support local farms and the local economy, improve nutritional quality, and lower the risk of chronic diseases.

With regard to adaptation, the health sector must engage in upgrading existing infrastructure and building workforce capacities to make health systems more climate resilient and contribute to climate and health literacy in communities. Further, the health sector can contribute by measuring carbon emission baselines and monitoring them on an ongoing basis, addressing sources of intense emission that include the use of eco-unfriendly anaesthetic gases, the use of coal-dependent electrification and the procurement of high carbon emitting goods.

Few adaptation measures take into account the special needs of women, infants, and children at different stages of development, as well as older persons with declines in capacities. Although significant attention has been focused on planning for health care systems to handle the crisis, including preparing for the increased frequency of extreme weather events, the preparation of the child care system and educational system or long-term care for older persons has received minimal attention. Monitoring water quality, indoor air quality, and crop yields will be essential to people-centred adaptations.

The Nurturing Care Framework [32] identified five components for children to survive, thrive, and transform health and human potential – good health, adequate nutrition, security and safety, responsive caregiving, and opportunities for early learning. Environmental health was described under the category of ‘security and safety’, but the impact of climate change was mentioned only in passing. Likewise, in well child and adolescent care, it has received little emphasis. The definition of environment used in describing age-friendly environments (‘all the factors in the extrinsic world that form the context of an individual’s life, including home, community and the broader society and factors in the environment that include the built environment, people and their relationships, attitudes and values, health and social policies, systems and services’) was silent about the natural environment [33]. Yet healthy natural environments underpin all life on earth. The built and natural environment is key in describing age-friendly environments, as noted in the World Health Organization (WHO) International Classification of Functioning, Disability and Health [34]. The time is right to consider a healthy natural environment as a core component of the enablers of health across the entire life course. A healthy environment is about much more than safety and security. Like nutrition, a healthy natural environment underpins well-being, growth and development, healthy reproduction and healthy ageing. Each of the three papers outline what more needs to be done to fill in evidence gaps considering the different climate hazards and outcomes beyond mortality.

### Climate action must include people of all ages

Mitigation, resilience-building and adaptation necessitate societal transformation, and such a transformation will require reassessing our values. It is important to reflect deeply on what societal and political values we embrace. Do we see it as our role to steward the natural environment for future generations? If so, traditional natural resource management can be revitalised. We can invoke sustainable lifestyles and move away from intense competition, achievement, individualism, and domination of nature. These have fuelled the climate crisis.

The world’s youth have taken the lead in highlighting the steps that individuals, families, communities, and society must now take. They have shown that it is possible to transform societal values and have given people of all ages reason for hope. To succeed, we will need to include embrace co-design with people from all life stages.

Each of us has a role to play in helping transform our world, and we should not linger on the sidelines but wholeheartedly join the efforts to mitigate and adapt to this existential crisis. Only then will this and future generations be able to fully embrace the human right to a clean, healthy and sustainable environment.

## References

[1] Conway F Portela A Filippi V Chou D Kovats S Climate change, air pollution and maternal and newborn health: An overview of reviews of health outcomes. J Glob Health. 2024;14:04128. 10.7189/jogh.14.04128 PMC11117177 38785109

[2] Proulx K Daelmans B Baltag V Banati P Climate change impacts on child and adolescent health and wellbeing: A narrative review. J Glob Health. 2024;14:04061. 38781568 10.7189/jogh.14.04061 PMC11115477

[3] PrinaMKhanNAkhter KanS.Healthy ageing and climate change: An assessment of the impact of climate hazards on older people. J Glob Health. 2024;14:04101.10.7189/jogh.14.0410138783708

[4] Romanello M di Napoli C Green C Kennard H Lampard P Scamman D The 2023 report of the Lancet Countdown on health and climate change: the imperative for a health-centred response in a world facing irreversible harms. Lancet. 2023;402:2346–94. 10.1016/S0140-6736(23)01859-7 37977174 PMC7616810

[5] Mycoo M, Wairiu M, Campbell D, Duvat V, Golbuu Y, Maharaj S, et al. Small Islands. In: Pörtner HO, Roberts DC, Tignor M, Poloczanska ES, Mintenbeck K, Alegria A, Craig M, Langsdorf S, Löschke S, Möller V, Okem A, Rama B, editors. Climate Change 2022: Impacts, Adaptation and Vulnerability. Contribution of Working Group II to the Sixth Assessment Report of the Intergovernmental Panel on Climate Change. Cambridge, UK: Cambridge University Press; 2022. p. 2043–2121. Available: https://www.ipcc.ch/report/ar6/wg2/downloads/report/IPCC_AR6_WGII_Chapter15.pdf. Accessed: 6 March 2024.

[6] Nazrul Islam S, Winkel J. Climate Change and Social Inequality. Department of Economic and Social Affairs Working Paper No. 152 ST/ESA/2017/DWP/152. 2017. Available: https://www.un.org/en/desa/climate-change-and-social-inequality. Accessed: 24 February 2024.

[7] Thacker S, Adshead D, Fantini C, Palmer R, Ghosal R, Adeoti T, et al. Infrastructure for climate action. Copenhagen, Denmark: United Nations Office for Project Services; 2021. Available: https://www.unops.org/news-and-stories/news/infrastructure-for-climate-action. Accessed: 24 February 2024.

[8] World Health Organization. The Right to Health. Geneva: World Health Organization; 2008. Available: https://www.ohchr.org/sites/default/files/Documents/Publications/Factsheet31.pdf. Accessed: 28 January 2024.

[9] United Nations General Assembly. Resolution adopted by the General Assembly on 25 September 2015. Transforming our World: The 2030 Agenda for Sustainable Development. USA: United Nations General Assembly; 2015. Available: https://documents.un.org/doc/undoc/gen/n15/291/89/pdf/n1529189.pdf?token=GsFXLuz1esJhHRuIrv&fe=true. Accessed: 28 January 2024.

[10] United Nations. Paris Agreement. Paris: United Nations; 2015. Available: https://unfccc.int/sites/default/files/english_paris_agreement.pdf. Accessed: 28 January 2024.

[11] Friel S Climate change mitigation: tackling the commercial determinants of planetary health inequity. Lancet. 2023;402:2269–71. 10.1016/S0140-6736(23)02512-6 37977168

[12] Romanello M Di Napoli C Drummond P Green C Kennard H Lampard P The 2022 report of the Lancet countdown on health and climate change: health at the mercy of fossil fuels. Lancet. 2022;400:1619–54. 10.1016/S0140-6736(22)01540-9 36306815 PMC7616806

[13] Yadav A Pacheco SE Prebirth effects of climate change on children’s respiratory health. Curr Opin Pediatr. 2023;35:344–9. 10.1097/MOP.0000000000001241 36974440

[14] Chersich MF Pham MD Areal A Haghighi M Manyuchi A Swift C Associations between high temperatures in pregnancy and risk of preterm birth, low birth weight and stillbirths: systematic review and meta-analysis. BMJ. 2020;371:m3811. 10.1136/bmj.m3811 33148618 PMC7610201

[15] Zhao Q Kress S Markevych I Berdel D von Berg A Gappa M Air pollution during infancy and lung function development into adolescence: The GINIplus/LISA birth cohorts study. Environ Int. 2021;146:106195. 10.1016/j.envint.2020.106195 33099064

[16] Pacheco SE Guidos-Fogelbach G Annesi-Maesano I Pawankar R D’Amato G Latour-Staffeld P American Academy of Allergy, Asthma & Immunology Environmental Exposures and Respiratory Health Committee. Climate change and global issues in allergy and immunology. J Allergy Clin Immunol. 2021;148:1366–77. 10.1016/j.jaci.2021.10.011 34688774

[17] World Health Organization. Mental Health and Climate Change: Policy Brief. Geneva: World Health Organization; 2022. Available: https://www.who.int/publications/i/item/9789240045125. Accessed: 24 February 2024.

[18] Maclean JC Popovici I French MT Are natural disasters in early childhood associated with mental health and substance use disorders as an adult? Soc Sci Med. 2016;151:78–91. 10.1016/j.socscimed.2016.01.006 26789078

[19] Zuelsdorff M Limaye VS A Framework for Assessing the Effects of Climate Change on Dementia Risk and Burden. Gerontologist. 2024;64:gnad082. 10.1093/geront/gnad082 37392416 PMC10860581

[20] Felitti VJ Anda RF Nordenberg D Williamson DF Spitz AM Edwards V Relationship of childhood abuse and household dysfunction to many of the leading causes of death in adults. The Adverse Childhood Experiences (ACE) Study. Am J Prev Med. 1998;14:245–58. 10.1016/S0749-3797(98)00017-8 9635069

[21] Bhutta ZA Bhavnani S Betancourt TS Tomlinson M Patel V Adverse childhood experiences and lifelong health. Nat Med. 2023;29:1639–48. 10.1038/s41591-023-02426-0 37464047

[22] Nixon R. Slow Violence and the Environmentalism of the Poor. Cambridge, Massachusetts: Harvard University Press; 2011.

[23] Evans S. Which countries are historically responsible for climate change? 2021. Available: https://www.carbonbrief.org/analysis-which-countries-are-historically-responsible-for-climate-change/. Accessed: 28 January 2024.

[24] United Nations. The human right to a clean, healthy and sustainable environment: resolution/adopted by the General Assembly. USA: United Nations; 2022. Available: https://digitallibrary.un.org/record/3983329?ln=en. Accessed: 28 January 2024.

[25] Intergovernmental Panel on Climate Change. Global Warming of 1.5°C. 2018. Available: https://www.ipcc.ch/sr15/. Accessed: 28 January 2024.

[26] Intergovernmental Panel on Climate Change. Climate change 2023: Summary for Policymakers. Geneva: Intergovernmental Panel on Climate Change; 2023. Available: https://www.ipcc.ch/report/ar6/syr/downloads/report/IPCC_AR6_SYR_SPM.pdf. Accessed: 6 March 2024.

[27] World Health Organization. WHO guidance to protect health from climate change through health adaptation planning. Geneva: World Health Organization; 2014. Available: https://www.who.int/publications/i/item/9789241508001. Accessed: 24 February 2024.

[28] World Health Organization. Quality Criteria for Health National Adaptation Plans. Geneva: World Health Organization; 2021. Available: https://www.who.int/publications/i/item/9789240018983. Accessed: 28 January 2024.

[29] World Health Organization. Climate change and health: vulnerability and adaptation assessment. Geneva: World Health Organization; 2021. Available: https://www.who.int/publications/i/item/9789240036383. Accessed: 28 January 2024.

[30] Food and Agriculture Organization of the United Nations, World Health Organization. Sustainable Healthy Diets: guiding principles. Geneva: World Health Organization; 2019. Available: https://www.who.int/publications/i/item/9789241516648. Accessed: 28 January 2024.

[31] World Health Organization. Health in the green economy: health co-benefits of climate change mitigation - transport sector. Geneva: World Health Organization; 2012. Available: https://www.who.int/publications/i/item/9789241502917. Accessed: 28 January 2024.

[32] World Health Organization, United Nations Children’s Fund, World Bank Group. Nurturing care for early childhood development: a framework for helping children survive and thrive to transform health and human potential. Geneva: World Health Organization; 2018. Available: https://apps.who.int/iris/bitstream/handle/10665/272603/9789241514064-eng.pdf. Accessed 24 February 2024.

[33] World Health Organization. National programmes for age-friendly cities and communities: a guide. Geneva: World Health Organization; 2023. Available: https://iris.who.int/bitstream/handle/10665/366634/9789240068698-eng.pdf?sequence=1. Accessed: 28 January 2024.

[34] World Health Organization. International Classification of Functioning, Disability and Health. Geneva: World Health Organization; 2001. Available: https://www.who.int/standards/classifications/international-classification-of-functioning-disability-and-health. Accessed: 28 January 2024.

